# Bis{2-[(*E*)-(4-fluoro­benz­yl)imino­meth­yl]-6-meth­oxy­phenolato-κ^2^ *N*,*O* ^1^}nickel(II)

**DOI:** 10.1107/S1600536811025189

**Published:** 2011-07-02

**Authors:** Hadariah Bahron, Amalina Mohd Tajuddin, Wan Nazihah Wan Ibrahim, Madhukar Hemamalini, Hoong-Kun Fun

**Affiliations:** a Faculty of Applied Sciences, Universiti Teknologi MARA, 40450 Shah Alam, Selangor, Malaysia; b X-ray Crystallography Unit, School of Physics, Universiti Sains Malaysia, 11800 USM, Penang, Malaysia

## Abstract

In the title compound, [Ni(C_15_H_13_FNO_2_)_2_], the Ni^II^ atom is tetra­coordinated by two N atoms and two O atoms from two 2-[(4-fluoro­benz­yl)imino­meth­yl]-6-meth­oxy­phenolate ligands in a square-planar geometry. The two N atoms and two O atoms around the Ni^II^ atom are *trans* to each other, as the Ni^II^ atom lies on an inversion centre. In the fluoro­phenyl group, five C atoms and an F atom are disordered over two sets of positions of equal occupancy. In the crystal, the complex mol­ecules are linked *via* inter­molecular C—H⋯F hydrogen bonds, forming chains along [001].

## Related literature

For applications of Schiff base complexes, see: Arun *et al.* (2009 ▶); Bagihalli *et al.* (2008 ▶); Yamada (1999 ▶). For a related structure, see: Mohd Tajuddin *et al.* (2010 ▶). For the synthesis of the ligand, see: Bahron *et al.* (2007 ▶).
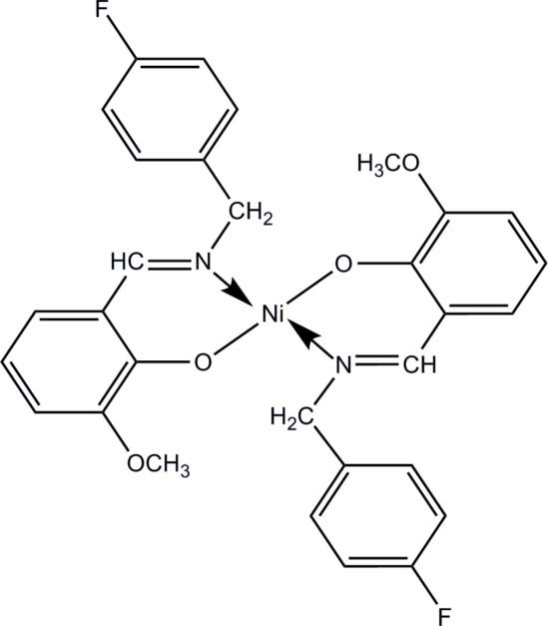


## Experimental

### 

#### Crystal data

[Ni(C_15_H_13_FNO_2_)_2_]
*M* *_r_* = 575.24
Triclinic, 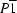

*a* = 5.0110 (2) Å
*b* = 10.9309 (4) Å
*c* = 12.2435 (5) Å
α = 109.631 (2)°
β = 99.083 (3)°
γ = 91.020 (3)°
*V* = 621.92 (4) Å^3^
*Z* = 1
Mo *K*α radiation
μ = 0.84 mm^−1^
*T* = 100 K
0.49 × 0.09 × 0.03 mm

#### Data collection

Bruker APEXII CCD diffractometer
Absorption correction: multi-scan (*SADABS*; Bruker, 2001 ▶) *T*_min_ = 0.685, *T*_max_ = 0.977
14720 measured reflections
3948 independent reflections
3148 reflections with *I* > 2σ(*I*)
*R*_int_ = 0.071

#### Refinement

*R*[*F* ^2^ > 2σ(*F* ^2^)] = 0.047
*wR*(*F* ^2^) = 0.114
*S* = 1.08
3948 reflections
222 parameters
H-atom parameters constrained
Δρ_max_ = 0.55 e Å^−3^
Δρ_min_ = −1.20 e Å^−3^

### 

Data collection: *APEX2* (Bruker, 2007 ▶); cell refinement: *SAINT* (Bruker, 2007 ▶); data reduction: *SAINT*; program(s) used to solve structure: *SHELXTL* (Sheldrick, 2008 ▶); program(s) used to refine structure: *SHELXTL*; molecular graphics: *SHELXTL*; software used to prepare material for publication: *SHELXTL* and *PLATON* (Spek, 2009 ▶).

## Supplementary Material

Crystal structure: contains datablock(s) global, I. DOI: 10.1107/S1600536811025189/hy2443sup1.cif

Structure factors: contains datablock(s) I. DOI: 10.1107/S1600536811025189/hy2443Isup2.hkl

Additional supplementary materials:  crystallographic information; 3D view; checkCIF report

## Figures and Tables

**Table 1:** Hydrogen-bond geometry (Å, °)

*D*—H⋯*A*	*D*—H	H⋯*A*	*D*⋯*A*	*D*—H⋯*A*
C5—H5*A*⋯F1^i^	0.95	2.51	3.363 (18)	150

Symmetry code: (i) 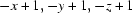.

**Fig. 1.**
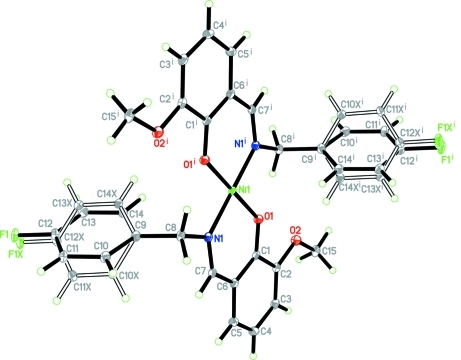
The molecular structure of the title compound, showing 30% probability displacement ellipsoids. [Symmetry code: (i) -x+1, -y+1, -z.]

**Fig. 2.**
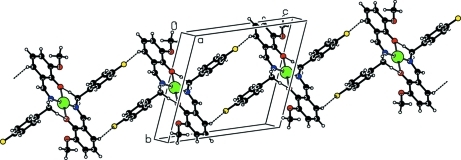
The crystal packing of the title compound. Only major components of the disordered atoms are shown. Dashed lines denote hydrogen bonds.

## supplementary crystallographic information

### Comment

Schiff base complexes are attractive due to their simple synthesis, versatility and diverse range of applications (Yamada, 1999). Nickel(II) Schiff base complexes have been reported to possess various properties such as anti-bacterial, anti-fungal (Bagihalli *et al.*, 2008) and catalytic activities (Arun *et al.*, 2009).  The title compound is bis-bidentate and related to the previously reported bis[2-(1-benzyliminoethyl)phenolato]palladium(II) (Mohd Tajuddin *et al.*, 2010), but with different metal centres and substituents on the iminoalkylphenolato and benzyl moieties.

The asymmetric unit has one half of the molecule, in which the Ni^II^ atom lies on an inversion centre (Fig. 1). The geometry around the Ni^II^ atom is square-planar, in which two N atoms and two O atoms are coplanar and *trans* to each other. The fluorophenyl group is disordered with five C atoms and an F atom over two sets of positions in an occupancy ratio of 0.50:0.50. The distances between the Ni atom and O and N atoms are 1.8298 (14) and 1.9223 (16) Å, respectively. In the crystal structure, the complex molecules are linked *via* C—H···F hydrogen bonds (Table 1, Fig. 2), forming a one-dimensional chain along [0 0 1].

### Experimental

The ligand 2-[(*E*)-(4-fluorobenzylimino)methyl]-6-methoxyphenol (0.519 g, 2 mmol), which was prepared according to the previously published method (Bahron *et al.*, 2007), was dissolved in ethanol (5 ml) in a round-bottomed flask. Nickel(II) acetate ( 0.251 g, 1 mmol) was dissolved separately in ethanol (5 ml ) and added into the flask containing the ligand solution. The mixture was stirred and refluxed for 5 h, upon which a green precipitate was formed. It was isolated by gravity filtration, washed with cold ethanol and air dried at room temperature. The solid product was recrystallized from chloroform, yielding green crystals (yield: 83.9%). Melting point: 493–498 K. Analysis, calculated for C_30_H_26_F_2_N_2_NiO_4_: C 62.64, H 4.56, N 4.87%; found: C 62.83, H 4.61, N 4.62%. IR (cm^-1^): ν(C═N) 1612 (s), ν(C–O) 1249 (s), ν(C–H) 2837 (w), ν(C–N) 1340 (m), ν(C–OCH_3_) 1082 (m), ν(Ni–O) 648 (w), ν(Ni–N) 462 (w). ^1^H NMR (CDCl_3_, 300 MHz, p.p.m.): δ = 6.238 (1H, s, HC═N), 6.741–7.559 (7H, m, H–aromatic), 5.695 (2H, s, CH_2_), 3.703 (3H, s, OCH_3_).

### Refinement

H atoms were positioned geometrically and refined using a riding model, with C—H = 0.95 (CH), 0.99 (CH_2_) and 0.98 (CH_3_) Å and with *U*_iso_(H) = 1.2(1.5 for methyl)*U*_eq_(C). Five C atoms and an F atom of the fluorophenyl group are disordered each over two sites, with a refined occupancy ratio of 0.507 (5):0.493 (5). In the final refinement, the occupancy ratio was fixed at 0.50:0.50.

### Crystal data

**Table:**

[Ni(C_15_H_13_FNO_2_)_2_]	*Z* = 1
*M**_r_* = 575.24	*F*(000) = 298
Triclinic, *P*1	*D*_x_ = 1.536 Mg m^−^^3^
Hall symbol: -P 1	Mo *K*α radiation, λ = 0.71073 Å
*a* = 5.0110 (2) Å	Cell parameters from 3312 reflections
*b* = 10.9309 (4) Å	θ = 3.1–29.9°
*c* = 12.2435 (5) Å	µ = 0.84 mm^−^^1^
α = 109.631 (2)°	*T* = 100 K
β = 99.083 (3)°	Needle, green
γ = 91.020 (3)°	0.49 × 0.09 × 0.03 mm
*V* = 621.92 (4) Å^3^	

### Data collection

**Table:**

Bruker APEXII CCD diffractometer	3948 independent reflections
Radiation source: fine-focus sealed tube	3148 reflections with *I* > 2σ(*I*)
graphite	*R*_int_ = 0.071
φ and ω scans	θ_max_ = 31.0°, θ_min_ = 1.8°
Absorption correction: multi-scan (*SADABS*; Bruker, 2001)	*h* = −7→7
*T*_min_ = 0.685, *T*_max_ = 0.977	*k* = −15→15
14720 measured reflections	*l* = −17→16

### Refinement

**Table:**

Refinement on *F*^2^	Primary atom site location: structure-invariant direct methods
Least-squares matrix: full	Secondary atom site location: difference Fourier map
*R*[*F*^2^ > 2σ(*F*^2^)] = 0.047	Hydrogen site location: inferred from neighbouring sites
*wR*(*F*^2^) = 0.114	H-atom parameters constrained
*S* = 1.08	*w* = 1/[σ^2^(*F*_o_^2^) + (0.0469*P*)^2^ + 0.2521*P*] where *P* = (*F*_o_^2^ + 2*F*_c_^2^)/3
3948 reflections	(Δ/σ)_max_ < 0.001
222 parameters	Δρ_max_ = 0.55 e Å^−^^3^
0 restraints	Δρ_min_ = −1.20 e Å^−^^3^

### Special details

**Table:**

Experimental. The crystal was placed in the cold stream of an Oxford Cryosystems Cobra open-flow nitrogen cryostat (Cosier, J. & Glazer, A. M. (1986). *J. Appl. Cryst.*19, 105–107) operating at 100.0 (1) K.

### Fractional atomic coordinates and isotropic or equivalent isotropic displacement parameters (Å^2^)

**Table:**

	*x*	*y*	*z*	*U*_iso_*/*U*_eq_	Occ. (<1)
Ni1	0.5000	0.5000	0.0000	0.01452 (11)	
O1	0.7199 (3)	0.64851 (13)	0.03573 (12)	0.0179 (3)	
O2	1.0870 (3)	0.82320 (14)	0.03948 (13)	0.0216 (3)	
N1	0.3601 (3)	0.56402 (15)	0.14468 (14)	0.0152 (3)	
C1	0.7786 (4)	0.74804 (18)	0.13306 (17)	0.0156 (4)	
C2	0.9789 (4)	0.84687 (19)	0.13974 (18)	0.0169 (4)	
C3	1.0462 (5)	0.95465 (19)	0.24012 (19)	0.0200 (4)	
H3A	1.1799	1.0191	0.2433	0.024*	
C4	0.9191 (5)	0.9703 (2)	0.33805 (19)	0.0221 (4)	
H4A	0.9681	1.0448	0.4071	0.027*	
C5	0.7253 (5)	0.87862 (19)	0.33413 (18)	0.0204 (4)	
H5A	0.6385	0.8902	0.4001	0.024*	
C6	0.6530 (4)	0.76618 (18)	0.23190 (18)	0.0170 (4)	
C7	0.4439 (4)	0.67372 (19)	0.22852 (17)	0.0162 (4)	
H7A	0.3562	0.6949	0.2952	0.019*	
C8	0.1401 (4)	0.48995 (19)	0.17120 (17)	0.0168 (4)	
H8A	0.0373	0.4283	0.0966	0.020*	
H8B	0.0131	0.5514	0.2102	0.020*	
C9	0.2526 (4)	0.41510 (19)	0.25017 (18)	0.0177 (4)	
C15	1.2842 (5)	0.9193 (2)	0.0404 (2)	0.0233 (4)	
H15A	1.3408	0.8945	−0.0367	0.035*	
H15B	1.4419	0.9256	0.1012	0.035*	
H15C	1.2057	1.0037	0.0574	0.035*	
F1	0.549 (4)	0.2029 (11)	0.4639 (17)	0.031 (2)	0.50
C10	0.1167 (9)	0.4015 (4)	0.3345 (4)	0.0197 (8)	0.50
H10A	−0.0475	0.4425	0.3450	0.024*	0.50
C11	0.2113 (10)	0.3290 (4)	0.4062 (4)	0.0228 (8)	0.50
H11A	0.1147	0.3206	0.4644	0.027*	0.50
C12	0.449 (2)	0.2710 (7)	0.3888 (8)	0.0228 (8)	0.50
C13	0.5903 (14)	0.2752 (6)	0.3032 (6)	0.0216 (12)	0.50
H13A	0.7511	0.2312	0.2925	0.026*	0.50
C14	0.4904 (12)	0.3462 (5)	0.2325 (5)	0.0197 (10)	0.50
H14A	0.5823	0.3489	0.1710	0.024*	0.50
F1X	0.583 (4)	0.2322 (11)	0.4752 (17)	0.0270 (16)	0.50
C10X	0.2322 (10)	0.4664 (4)	0.3718 (4)	0.0200 (8)	0.50
H10B	0.1442	0.5442	0.4006	0.024*	0.50
C11X	0.3399 (9)	0.4039 (4)	0.4485 (4)	0.0214 (7)	0.50
H11B	0.3243	0.4367	0.5294	0.026*	0.50
C12X	0.471 (2)	0.2918 (7)	0.4034 (8)	0.0214 (7)	0.50
C13X	0.4963 (13)	0.2427 (6)	0.2862 (6)	0.0201 (12)	0.50
H13B	0.5866	0.1657	0.2571	0.024*	0.50
C14X	0.3865 (11)	0.3080 (5)	0.2107 (5)	0.0164 (9)	0.50
H14B	0.4071	0.2760	0.1303	0.020*	0.50

### Atomic displacement parameters (Å^2^)

**Table:**

	*U*^11^	*U*^22^	*U*^33^	*U*^12^	*U*^13^	*U*^23^
Ni1	0.0169 (2)	0.01101 (16)	0.01719 (18)	−0.00043 (13)	0.00410 (14)	0.00644 (13)
O1	0.0213 (8)	0.0145 (6)	0.0180 (7)	−0.0027 (6)	0.0053 (6)	0.0050 (5)
O2	0.0236 (8)	0.0186 (7)	0.0237 (7)	−0.0048 (6)	0.0078 (6)	0.0073 (6)
N1	0.0152 (8)	0.0143 (7)	0.0171 (8)	−0.0003 (6)	0.0027 (6)	0.0069 (6)
C1	0.0165 (10)	0.0120 (8)	0.0189 (9)	0.0018 (7)	0.0009 (7)	0.0070 (7)
C2	0.0159 (10)	0.0160 (8)	0.0209 (9)	0.0020 (7)	0.0030 (8)	0.0092 (7)
C3	0.0200 (10)	0.0163 (8)	0.0247 (10)	−0.0018 (8)	0.0024 (8)	0.0091 (8)
C4	0.0268 (12)	0.0152 (9)	0.0208 (10)	−0.0034 (8)	0.0020 (9)	0.0027 (8)
C5	0.0238 (11)	0.0174 (9)	0.0191 (9)	0.0010 (8)	0.0049 (8)	0.0046 (8)
C6	0.0189 (10)	0.0141 (8)	0.0192 (9)	0.0007 (7)	0.0030 (8)	0.0073 (7)
C7	0.0179 (10)	0.0164 (8)	0.0168 (9)	0.0034 (7)	0.0044 (7)	0.0079 (7)
C8	0.0147 (9)	0.0171 (8)	0.0208 (9)	0.0007 (7)	0.0043 (8)	0.0090 (7)
C9	0.0169 (10)	0.0171 (8)	0.0203 (9)	−0.0024 (7)	0.0036 (8)	0.0083 (7)
C15	0.0204 (11)	0.0216 (9)	0.0333 (12)	−0.0020 (8)	0.0070 (9)	0.0155 (9)
F1	0.050 (6)	0.024 (4)	0.030 (4)	0.009 (4)	0.008 (3)	0.021 (4)
C10	0.020 (2)	0.0161 (18)	0.025 (2)	−0.0036 (17)	0.0072 (17)	0.0072 (16)
C11	0.032 (2)	0.0187 (16)	0.0222 (19)	0.0007 (15)	0.0068 (16)	0.0117 (14)
C12	0.032 (2)	0.0187 (16)	0.0222 (19)	0.0007 (15)	0.0068 (16)	0.0117 (14)
C13	0.024 (3)	0.018 (3)	0.023 (3)	0.006 (2)	0.005 (3)	0.008 (2)
C14	0.024 (3)	0.017 (2)	0.019 (2)	0.0003 (19)	0.006 (2)	0.0069 (18)
F1X	0.037 (4)	0.026 (5)	0.028 (3)	0.006 (4)	0.009 (3)	0.020 (4)
C10X	0.023 (2)	0.0192 (19)	0.0190 (19)	0.0064 (18)	0.0054 (17)	0.0076 (16)
C11X	0.0254 (19)	0.0235 (18)	0.0163 (17)	0.0022 (15)	0.0007 (14)	0.0096 (14)
C12X	0.0254 (19)	0.0235 (18)	0.0163 (17)	0.0022 (15)	0.0007 (14)	0.0096 (14)
C13X	0.026 (3)	0.016 (3)	0.020 (3)	0.003 (2)	0.006 (3)	0.007 (2)
C14X	0.020 (2)	0.013 (2)	0.018 (2)	0.0003 (17)	0.0049 (19)	0.0067 (17)

### Geometric parameters (Å, °)

**Table:**

Ni1—O1	1.8297 (14)	C9—C10X	1.425 (5)
Ni1—N1	1.9223 (16)	C9—C14	1.428 (6)
O1—C1	1.303 (2)	C15—H15A	0.9800
O2—C2	1.367 (2)	C15—H15B	0.9800
O2—C15	1.425 (2)	C15—H15C	0.9800
N1—C7	1.299 (2)	F1—C12	1.41 (2)
N1—C8	1.495 (2)	C10—C11	1.404 (6)
C1—C6	1.409 (3)	C10—H10A	0.9500
C1—C2	1.434 (3)	C11—C12	1.371 (12)
C2—C3	1.376 (3)	C11—H11A	0.9500
C3—C4	1.407 (3)	C12—C13	1.367 (13)
C3—H3A	0.9500	C13—C14	1.390 (9)
C4—C5	1.367 (3)	C13—H13A	0.9500
C4—H4A	0.9500	C14—H14A	0.9500
C5—C6	1.420 (3)	F1X—C12X	1.32 (2)
C5—H5A	0.9500	C10X—C11X	1.388 (6)
C6—C7	1.429 (3)	C10X—H10B	0.9500
C7—H7A	0.9500	C11X—C12X	1.389 (10)
C8—C9	1.516 (3)	C11X—H11B	0.9500
C8—H8A	0.9900	C12X—C13X	1.380 (12)
C8—H8B	0.9900	C13X—C14X	1.402 (9)
C9—C14X	1.344 (5)	C13X—H13B	0.9500
C9—C10	1.369 (5)	C14X—H14B	0.9500
			
O1—Ni1—O1^i^	180.00 (9)	C10X—C9—C14	111.5 (3)
O1—Ni1—N1	93.00 (7)	C14X—C9—C8	122.0 (3)
O1^i^—Ni1—N1	87.00 (7)	C10—C9—C8	121.1 (3)
O1—Ni1—N1^i^	87.00 (7)	C10X—C9—C8	118.4 (2)
O1^i^—Ni1—N1^i^	93.00 (6)	C14—C9—C8	121.3 (3)
N1—Ni1—N1^i^	180.0	O2—C15—H15A	109.5
C1—O1—Ni1	130.38 (13)	O2—C15—H15B	109.5
C2—O2—C15	116.49 (16)	H15A—C15—H15B	109.5
C7—N1—C8	113.22 (16)	O2—C15—H15C	109.5
C7—N1—Ni1	124.50 (14)	H15A—C15—H15C	109.5
C8—N1—Ni1	122.29 (12)	H15B—C15—H15C	109.5
O1—C1—C6	123.93 (18)	C9—C10—C11	122.4 (4)
O1—C1—C2	118.37 (18)	C9—C10—H10A	118.8
C6—C1—C2	117.68 (17)	C11—C10—H10A	118.8
O2—C2—C3	125.29 (19)	C12—C11—C10	117.3 (6)
O2—C2—C1	114.03 (17)	C12—C11—H11A	121.3
C3—C2—C1	120.68 (19)	C10—C11—H11A	121.3
C2—C3—C4	120.63 (19)	C13—C12—C11	123.9 (9)
C2—C3—H3A	119.7	C13—C12—F1	118.8 (12)
C4—C3—H3A	119.7	C11—C12—F1	117.3 (12)
C5—C4—C3	120.18 (19)	C12—C13—C14	117.6 (7)
C5—C4—H4A	119.9	C12—C13—H13A	121.2
C3—C4—H4A	119.9	C14—C13—H13A	121.2
C4—C5—C6	120.33 (19)	C13—C14—C9	121.4 (5)
C4—C5—H5A	119.8	C13—C14—H14A	119.3
C6—C5—H5A	119.8	C9—C14—H14A	119.3
C1—C6—C5	120.49 (19)	C11X—C10X—C9	120.6 (3)
C1—C6—C7	120.17 (18)	C11X—C10X—H10B	119.7
C5—C6—C7	119.30 (18)	C9—C10X—H10B	119.7
N1—C7—C6	127.39 (18)	C10X—C11X—C12X	118.0 (5)
N1—C7—H7A	116.3	C10X—C11X—H11B	121.0
C6—C7—H7A	116.3	C12X—C11X—H11B	121.0
N1—C8—C9	111.72 (16)	F1X—C12X—C13X	118.8 (11)
N1—C8—H8A	109.3	F1X—C12X—C11X	119.3 (10)
C9—C8—H8A	109.3	C13X—C12X—C11X	121.9 (8)
N1—C8—H8B	109.3	C12X—C13X—C14X	119.0 (7)
C9—C8—H8B	109.3	C12X—C13X—H13B	120.5
H8A—C8—H8B	107.9	C14X—C13X—H13B	120.5
C14X—C9—C10	108.2 (3)	C9—C14X—C13X	121.0 (5)
C14X—C9—C10X	119.5 (3)	C9—C14X—H14B	119.5
C10—C9—C14	117.3 (3)	C13X—C14X—H14B	119.5
			
N1—Ni1—O1—C1	8.77 (19)	N1—C8—C9—C10	143.9 (3)
N1^i^—Ni1—O1—C1	−171.23 (19)	N1—C8—C9—C10X	102.8 (3)
O1—Ni1—N1—C7	−4.67 (18)	N1—C8—C9—C14	−42.0 (3)
O1^i^—Ni1—N1—C7	175.33 (18)	C14X—C9—C10—C11	29.7 (5)
O1—Ni1—N1—C8	175.40 (15)	C10X—C9—C10—C11	−85.9 (6)
O1^i^—Ni1—N1—C8	−4.60 (15)	C14—C9—C10—C11	3.4 (6)
Ni1—O1—C1—C6	−7.0 (3)	C8—C9—C10—C11	177.8 (3)
Ni1—O1—C1—C2	174.36 (14)	C9—C10—C11—C12	0.0 (7)
C15—O2—C2—C3	−0.3 (3)	C10—C11—C12—C13	−2.9 (9)
C15—O2—C2—C1	179.05 (18)	C10—C11—C12—F1	177.4 (7)
O1—C1—C2—O2	−0.1 (3)	C11—C12—C13—C14	2.1 (10)
C6—C1—C2—O2	−178.77 (18)	F1—C12—C13—C14	−178.2 (7)
O1—C1—C2—C3	179.33 (19)	C12—C13—C14—C9	1.6 (8)
C6—C1—C2—C3	0.6 (3)	C14X—C9—C14—C13	−79.1 (9)
O2—C2—C3—C4	179.04 (19)	C10—C9—C14—C13	−4.2 (6)
C1—C2—C3—C4	−0.3 (3)	C10X—C9—C14—C13	34.5 (6)
C2—C3—C4—C5	−0.4 (3)	C8—C9—C14—C13	−178.6 (4)
C3—C4—C5—C6	0.8 (3)	C14X—C9—C10X—C11X	−2.6 (6)
O1—C1—C6—C5	−178.92 (19)	C10—C9—C10X—C11X	77.3 (5)
C2—C1—C6—C5	−0.3 (3)	C14—C9—C10X—C11X	−29.9 (5)
O1—C1—C6—C7	−1.2 (3)	C8—C9—C10X—C11X	−177.9 (3)
C2—C1—C6—C7	177.37 (18)	C9—C10X—C11X—C12X	1.1 (8)
C4—C5—C6—C1	−0.4 (3)	C10X—C11X—C12X—F1X	178.0 (9)
C4—C5—C6—C7	−178.1 (2)	C10X—C11X—C12X—C13X	0.1 (10)
C8—N1—C7—C6	179.02 (19)	F1X—C12X—C13X—C14X	−177.8 (9)
Ni1—N1—C7—C6	−0.9 (3)	C11X—C12X—C13X—C14X	0.1 (11)
C1—C6—C7—N1	5.2 (3)	C10—C9—C14X—C13X	−34.3 (6)
C5—C6—C7—N1	−177.1 (2)	C10X—C9—C14X—C13X	2.8 (7)
C7—N1—C8—C9	−80.9 (2)	C14—C9—C14X—C13X	81.2 (9)
Ni1—N1—C8—C9	99.07 (17)	C8—C9—C14X—C13X	177.9 (4)
N1—C8—C9—C14X	−72.4 (3)	C12X—C13X—C14X—C9	−1.6 (9)

Symmetry codes: (i) −*x*+1, −*y*+1, −*z*.

### Hydrogen-bond geometry (Å, °)

**Table:**

*D*—H···*A*	*D*—H	H···*A*	*D*···*A*	*D*—H···*A*
C5—H5A···F1^ii^	0.95	2.51	3.363 (18)	150

Symmetry codes: (ii) −*x*+1, −*y*+1, −*z*+1.

## References

[bb1] Arun, V., Sridevi, N., Robinson, P. P., Manju, S. & Yusuff, K. K. M. (2009). *J. Mol. Catal. A Chem.* **304**, 191–198.

[bb2] Bagihalli, G. B., Avaji, P. G., Patil, S. A. & Badami, P. S. (2008). *Eur. J. Med. Chem.* **43**, 2639–2649. 10.1016/j.ejmech.2008.02.013 18395942

[bb3] Bahron, H., Kassim, K., Omar, S. R. S., Rashid, S. H., Fun, H.-K. & Chantrapromma, S. (2007). *Acta Cryst.* E**63**, o558–o560.

[bb4] Bruker (2001). *SADABS* Bruker AXS Inc., Madison, Wisconsin, USA.

[bb5] Bruker (2007). *APEX2* and *SAINT* Bruker AXS Inc., Madison, Wisconsin, USA.

[bb6] Mohd Tajuddin, A., Bahron, H., Wan Ibrahim, W. N. & Yamin, B. M. (2010). *Acta Cryst.* E**66**, m1100. 10.1107/S1600536810031375 PMC3007840 21588512

[bb7] Sheldrick, G. M. (2008). *Acta Cryst.* A**64**, 112–122. 10.1107/S0108767307043930 18156677

[bb8] Spek, A. L. (2009). *Acta Cryst.* D**65**, 148–155. 10.1107/S090744490804362X PMC2631630 19171970

[bb9] Yamada, S. (1999). *Coord. Chem. Rev.* **190–192**, 537–555.

